# Failure Severity Prediction for Protective-Coating Disbondment via the Classification of Acoustic Emission Signals

**DOI:** 10.3390/s23156833

**Published:** 2023-07-31

**Authors:** Noor A’in A. Rahman, Zazilah May, Rabeea Jaffari, Mehwish Hanif

**Affiliations:** 1 Department of Electrical and Electronics Engineering, Universiti Teknologi PETRONAS, Seri Iskandar 32610, Malaysia; noor_20001814@utp.edu.my (N.A.A.R.); mehwish_16005841@utp.edu.my (M.H.); 2 High Performance Cloud Computing Centre, Universiti Teknologi PETRONAS, Seri Iskandar 32610, Malaysia; 3 Centre for System Engineering, Universiti Teknologi PETRONAS, Seri Iskandar 32610, Malaysia; 4 Software Engineering Department, Mehran University of Engineering and Technology, Jamshoro 76062, Pakistan; rabeea.jaffari@faculty.muet.edu.pk

**Keywords:** acoustic emission, coating disbondment, machine learning, classification

## Abstract

Structural health monitoring is a popular inspection method that utilizes acoustic emission (AE) signals for fault detection in engineering infrastructures. Diagnosis based on the propagation of AE signals along any surface material offers an attractive solution for fault identification. However, the classification of AE signals originating from failure events, especially coating failure (coating disbondment), is a challenging task given the AE signature of each material. Thus, different experimental settings and analyses of AE signals are required to classify the various types of coating failures, and they are time-consuming and expensive. Hence, to address these issues, we utilized machine learning (ML) classification models in this work to evaluate epoxy-based-protective-coating disbondment based on the AE principle. A coating disbondment experiment consisting of coated carbon steel test panels for the collection of AE signals was implemented. The obtained AE signals were then processed to construct the final dataset to train various state-of-the-art ML classification models to divide the failure severity of coating disbondment into three classes. Consequently, methods for the extraction of useful features, the handling of data imbalance, and a reduction in the bias of ML models were also effectively utilized in this study. Evaluations of state-of-the-art ML classification models on the AE signal dataset in terms of standard metrics revealed that the decision forest classification model outperformed the other state-of-the-art models, with accuracy, precision, recall, and F1 score values of 99.48%, 98.76%, 97.58%, and 98.17%, respectively. These results demonstrate the effectiveness of utilizing ML classification models for the failure severity prediction of protective-coating defects via AE signals.

## 1. Introduction

Coating failure is one of the major problems faced by various industries around the globe. A broad range of environmental factors during services and paint applications can result in coating failure and delamination. In general, coating failure occurs due to an electrochemical reaction beneath the coating. In aggressive coastal marine environments, the causes of coating failures of offshore pipelines can be divided into the following two categories: corrosion due to microorganism activity [1,2,3] and maritime activities, including wet–dry cyclic conditions such as those at splash zone areas [4], and other/unknown causes [5]. In the aerospace industry, exterior coating failure occurs due to the high temperatures of engine exhausts and the drag effect of atmospheric friction [6]. Coating failure in water tank/storage results from water seepage, abrasion, and solvent trapped under the coating film. Moreover, other common causes of coating failures are related to coating preparation, including improper or inadequate surface preparation, improper application environments, improper application techniques, and improper formulation [7]. Coating defects, which are unavoidable during the coating preparation stages, invite corrosion. To reduce the probability of corrosion occurrences, pipeline operators convert pipe spools into cathodes by applying a cathodic protection (CP) current to inhibit electron migration. Nevertheless, the “overprotection” provided by a CP current can lead to reaction products that influence coating adhesion and cause coating delamination, also known as cathodic delamination.

Severe coating failures involve operational integrity and safety issues that generate high maintenance costs. Structural health and monitoring (SHM) is becoming a reliable technique for inspecting the conditions of coatings in various structures. In the past several years, acoustic emission (AE) has been considered an SHM technique that is cost-effective in monitoring any type of metallic or [8,9,10,11] nonmetallic infrastructure. AE is a nondestructive testing technology that utilizes the energy-propagation-based monitoring technique. AE features are unique and generated from the stress waves captured during energy release. The feasibility of AE for use in monitoring the corrosion events of carbon–epoxy-coated aluminum was studied by D. Baltzis et al. [12]. According to their work, AE sensors detected the energy released by hydrogen bubble production during cathodic polarization and the deposition of thick soluble films during the final stage of anodic polarization. Thus, further research on the use of AE to monitor aluminum corrosion tools can be carried out. M. Sause et al. [13] speculated that the evolution of coating breakdown on nickel–copper-coated materials, which consists of crack initiation, crack growth, and delamination, can be distinguished from the pattern recognition of acquired AE signals. Another work with epoxy resin acting as coating on a metal substrate was carried out by Louda et al. [14]. The major drawback of AE was the complex and dynamic process involved in the analysis of AE output signals, especially in determining the most significant AE features. Despite numerous reports on the capability of AE to detect failures in metallic structures, a very minimal amount of research has focused on monitoring external coating failure, also known as disbondment. Achieving a reliable failure identification model for different coating systems is very challenging given the AE signal signature of each material. Thus, different settings and analyses of AE signals are required for the various types of coating failure. AE features, such as energy, amplitude, count rate, counts, signal strength, and rise time, are important features that have been considered in dealing with AE signal analysis.

In real applications, the extraction and classification of AE signal features due to coating disbondment contribute to pipeline integrity. However, the classification of AE signals originating from failure, especially coating disbondment, remains controversial. The reason for this controversy is the difficulty in sorting the AE signals caused by disbondment because other cathodic reaction products are also produced. ML is widely utilized by researchers and practitioners in various research areas, such as computer vision [15,16], speech recognition [17,18], mechanical engineering [19,20], civil engineering [21,22], and so on, with positive results. Similarly, a wide variety of research works have also utilized ML algorithms to classify the AE features generated from various monitoring events [23,24,25,26]. In [23], support vector machines (SVMs) were utilized on AE signal data for the fault detection and classification of ball-bearing components in rotary machines, and the researchers in [24] used a wavelet neural network to identify various corrosion AE signals from different types of atmospheric vertical storage tanks. The researchers in [25] utilized and tested various ML algorithms to classify AE features from different wear categories of a pin-on-disc tribometer in friction-related industrial processes, and the work in [26] developed a software called RF-CAM to classify AE signals from crevice corrosion experiments using random forest. None of the discussed research utilized ML for the failure classification of coating disbondment events. Moreover, apart from ML, a few [27,28] studies have utilized deep learning (DL) techniques on AE signal data to localize AE sources in common and complex metallic panels, with autoencoders used in [27] and for the detection and localization of cracks in steel rails under loads in [28]. However, DL techniques are often limited in their function due to their reliance on large training datasets, as discussed by the researchers in [29]. Hence, inspired by advancements in ML and AE signal processing and analysis, this work was undertaken to fill the existing research gaps by proposing an ML-based classification approach for coating disbondment failure severity prediction via AE features.

In this work, a disbondment signal identification model was developed by comparing values obtained from the classification analysis of various AE signals acquired from the same sources but in varying severities. In this regard, 15 AE features were utilized to identify the best model to classify AE signals during coating disbondment activities into three failure classes. This work used an epoxy-based coating to coat the specimen due to its capability and widely used sensors. Hence, the significance and contributions of the current work are as follows:

- The introduction of an overprotection phenomenon (based on voltage charging) to accelerate the coating disbondment process produced a challenging condition for the classification of coating disbondment severity.
- A novel AE dataset for future ML-based coating disbondment research. The AE data collected were divided into three classes based on the severity of failure corresponding to the charging voltage during the experiment. Each class was presented to provide a basis for the features and characterization of AE, originating from different severity levels, with 1 and 3 specifying the lowest and highest failure severities, respectively.
- A novel methodology utilizing the application and evaluation of various state-of-the-art machine learning (ML) classifiers on the collected AE data was used to predict the failure severity level of the coating disbondment event.

To the best of the authors’ knowledge, the methods utilized in this work are novel, with no application of ML to coating disbondment failure severity prediction to date. Moreover, this work is valuable because the results can help in making better choices for the inspection interval of external coatings with overprotection issues. The methodology in the current research can be extended and applied to other coating materials, and the formulated AE dataset can be utilized in future ML-based coating disbondment research.

## 2. AE Signal

**Figure 1:**
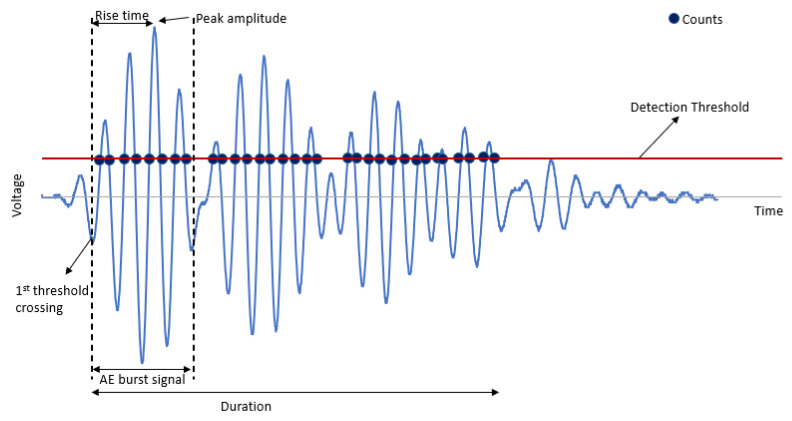
Several features of a typical AE signal/waveform.

Having an informative signal is essential to producing a reliable ML predictive model. The AE signal features were utilized to develop an ML classification model for the identification and classification of the failure severity of coating disbondment events. Several AE features from AE signals (also called waveforms) can be extracted via different methods, and they are utilized in failure detection in coating disbondments. Figure 1 shows the typical AE features contained in an AE waveform. In general, a waveform consists of amplitude, rise time, counts, and duration. The detection threshold is expressed on the decibel (dB) scale, and counts to peak refers to the number of thresholds crossing from the first to highest voltage points on the waveform. The second-order derivatives of AE features calculated from the area under the waveform include signal and energy.

## 3. Materials and Methods

**Figure 2:**
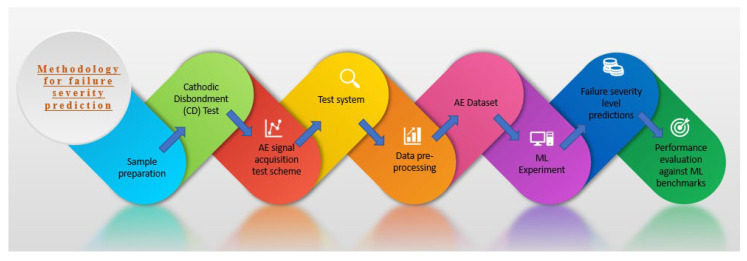
Methodology for failure severity prediction in coating disbondment event.

The coating failure signature from AE signals was obtained, and the coating failure was created in the laboratory. This section explains the experiment, data acquisition (DAQ), and data analysis of the given sample. A test sample prepared before coating failure was created on the sample to obtain the AE signature. The resulting experimental data were then utilized to constitute the final dataset for training the ML classification models. The complete methodology is depicted in Figure 2, and all the steps are described in the subsequent subsections (Section 3.1, Section 3.2, Section 3.3, Section 3.4, Section 3.5 and Section 3.6).

### 3.1. Sample Preparation

**Figure 3:**
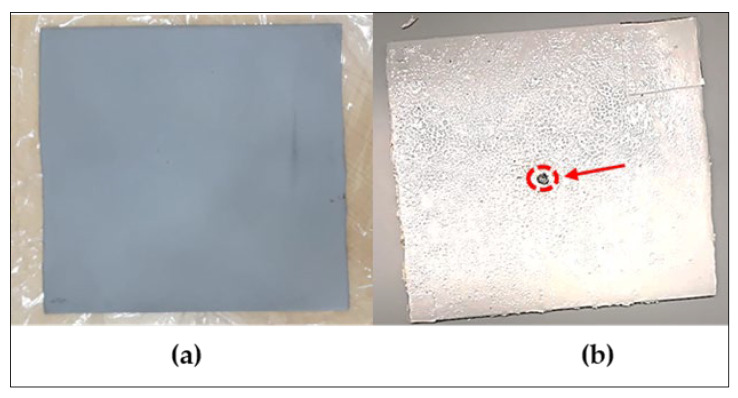
CD test panels: (**a**) test panel before coating; (**b**) test panel after coating. The red circle shows that the artificial defect was created once the coating was cured.

A carbon steel test panel of size 150 × 150 × 2 mm^3^ was prepared and grit-blasted to standard abrasive 2.5 to achieve a 50–55 µm anchor pattern. The substrate was coated with an epoxy-based coating as the primer and a topcoat and polyurethane coating as the secondary layer. The coating was applied using the spray technique. The dry film thickness was maintained below 200 µm for each substrate. The specimens were ready to be used once the curing time had elapsed (circa 24 h at room temperature). According to the American Society for Testing and Materials (ASTM) G8, an artificial defect needs to be created on the cathodic debonding (CD) test panel using a drill bit. The diameter of the defect was approximately 6.35 mm (Figure 3b).

### 3.2. CD Test

**Figure 4:**
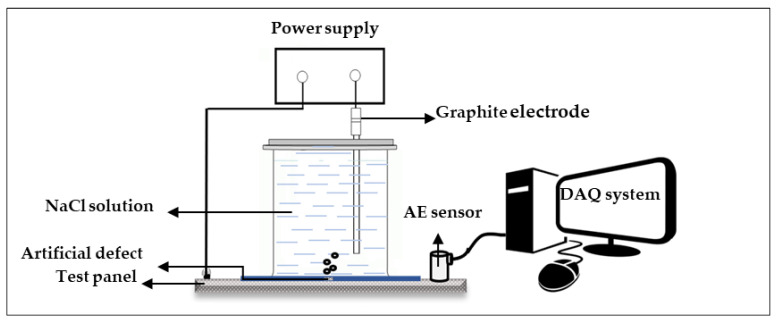
CD experimental setup consisting of a complete AE acquisition system and designated defect on the test panel.

Coating disbondment was initiated, where the three levels of overprotection condition were supplied by inducing a certain amount of current. Figure 4 shows the experimental platform. The experiments were performed following the ASTM G8 standard [20]. Each coated test panel was attached to a plastic cylinder container filled with 3.5 wt.% NaCl solution, which served as an electrolyte in this test. The coated test panel served as the working electrode, and a graphite electrode was used to complete the circuit for this test. Voltages of 3.0, 4.5, and 6.0 V were applied with a DC power supply on the panels to perform the disbondment event for 24 h each. This action is called a “charging” event from this point onward. The AE signals were generated from the sound of the disbonded coating event and recorded by the AE sensor (R1.5I). The AE sensor was secured on the test panel by a magnetic clamp. Furthermore, a silicon-based coupling agent was used to obtain smooth signal transfer between the test panel and sensor. Skipping this step resulted in partial signal loss. The AE signal was monitored using the single-datum collection channels of a DAQ system. Physical Acoustics Corporation (PAC) (USA) supplied the entire DAQ system, including the sensor and signaling cable.

### 3.3. Test System

The R1.5I sensor, which is a type of AE sensor, was provided by PAC, with a 5 kHz to 20 kHz operating frequency and −35 °C to +75 °C operating temperatures. The sensor was used to capture the breakdown signal (elastic wave) propagated on the substrate with a shock limit equal to 500 g. The AE sensor was a piezoelectric material-based sensor that converts elastic waves in carbon steel substrates into an electrical AE signal. The AE signal was then captured and carried through a signaling coaxial cable and converted using an analog-to-digital converter. To acquire the raw AE signal, we used high-performance data sampling and appropriate DAQ. This work used the module equipped with AEwin software [30], Express-8 Version V5.92, provided by PAC. Details on the sensor are shown in Table 1.

### 3.4. AE Signal Acquisition Test Scheme

**Figure 5:**
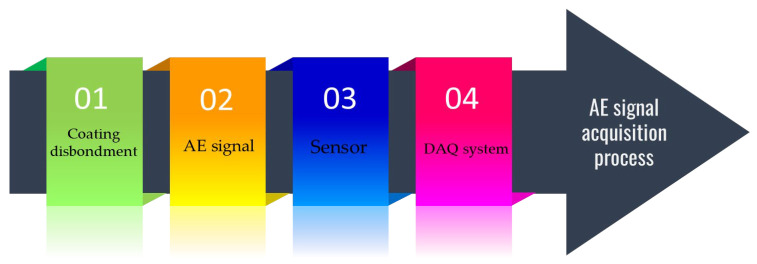
AE signal acquisition process.

Figure 5 depicts the AE signal acquisition process. Before and during the experiment, data on the background (before starting the CD test) and during the CD tests were collected at a sampling rate of 1 µs per sample.

The following were the procedures employed to measure the AE signal during the CD test:

- Sensor installation: A coupling agent (silicon-based) was applied at the bottom of the AE sensor. Then, the sensor was adhered to the uncoated surface area. The surface area was kept smooth and clean prior to sensor installation.
- Prior to AE signal acquisition, the environmental noise level was assessed. This step helped in determining the appropriate threshold for AE signal acquisition. A noise assessment was performed by conducting test acquisition for several minutes and analyzing the amplitude distribution range. The threshold amplitude was set above the ambient noise level for all tests in the acquisition setting.
- Conducting the pencil lead break (PLB) test: Three PLB tests were conducted near the break point of each sensor to vary their accuracy and connection. The PLB test was used to produce the standard AE signals by employing a hard black lead core with a diameter of 0.5 mm and a pen core extension length of 2.5 mm. The lead core was broken at a selected point at a 30° angle to the surface of the test panel to comply with PLB testing. This test was also applied to check the sensitivity of the sensor. The PLB test was performed in accordance with the ASTM E-976 standards and specifications [31].
- AE signal acquisition: the AE signal acquisition was monitored and recorded for 15 min each hour (a total of 24 h for each test set) toward the end of the experiment.
- A visual examination was immediately performed at the end of the test, and the disbondment area was calculated and recorded.

### 3.5. Data Preprocessing

**Figure 6:**
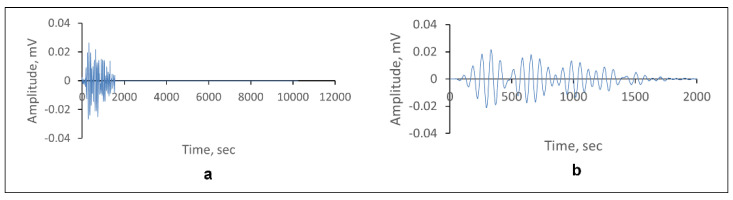
Example of: (**a**) a raw waveform; (**b**) a waveform after preprocessing.

During the coating disbondment and failure, the AE source was released mainly by byproduct reaction. Thus, a filtering process must be performed prior to any further analysis. The AE hit generated a 10024-line set per waveform. The AE features were generated internally from the waveform by the AEwin software based on the calculation of the waveform adapted during acquisition. Signal preprocessing included waveform tail cutting and the removal of hits that had zero energy, while preserving the information in the original dataset through the process. Figure 6 shows examples of waveforms before and after preprocessing. The cleaned waveforms were used to generate the final AE feature dataset to be fed to the ML model for training.

#### AE Dataset

The entire AE dataset was acquired from the CD test. The generation of the AE dataset solely depended on the disbondment activity and predefined threshold. The threshold value was set at 45 dB in the CD test for 24 h. The high volume of the signal recorded during the activity was due to the coating disbondment and production of hydrogen bubbles during the charging process. The AE data were categorized into Classes 1 (charging voltage: 3.0 V), 2 (charging voltage: 4.5 V), and 3 (charging voltage: 6.0 V) based on the voltage applied during the charging activity. These classes signify the failure severity levels, with Classes 1, 2, and 3 being the lowest, medium, and most severe, respectively. In total, 203,711 waveforms were generated after preprocessing. The AE features were generated from the waveforms for each class. Table 2 describes the AE features used in this study along with their units.

**Figure 7:**
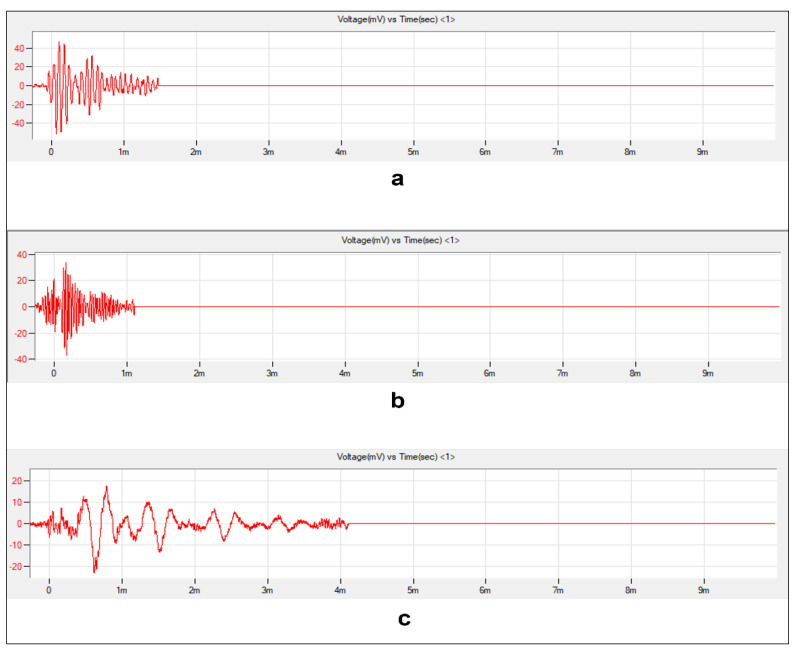
Typical AE waveform of each class: (**a**) Class 1, (**b**) Class 2, and (**c**) Class 3.

Given that the severity class of the coating disbondment failure was to be predicted, the problem was framed as a supervised ML classification task with the abovementioned 15 AE features as input variables and the severity class (1–3) as the target variable to be predicted. The final AE dataset was normalized to ensure data uniformity in the ML experiment. In total, we observed 15 AE feature variables, with each containing 203,708 samples, and one target variable belonging to classes 1, 2, and 3, which signify the lowest, medium, and the most severe failure levels, respectively. Figure 7 depicts the typical AE waveform of each class.

### 3.6. ML Experiment

**Figure 8:**
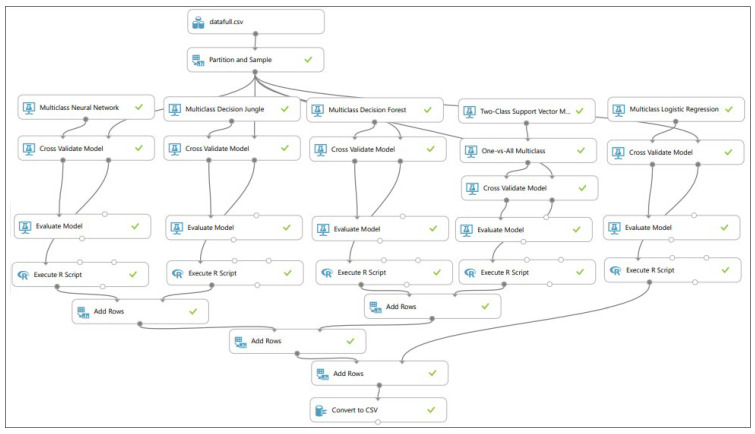
ML experiment for failure severity prediction in coating disbondment event.

The ML experiment on the prediction of the failure severity class for the coating disbondment event based on the constructed AE dataset was carried out using Microsoft Azure Machine Learning Studio [22]. Five state-of-the-art ML multiclass classification models, namely, the neural network, decision jungle, decision forest, SVM, and logistic regression, were trained and validated on the AE dataset using k-fold (k = 10) cross validation [23], where the 203,708 samples in the dataset were split into 10 folds with 9 folds being used for training and the remaining 10th fold used for validating the models. k-Fold cross validation was utilized for training and validation to prevent overfitting bias, if any, in the dataset. Moreover, stratified data sampling was utilized to handle the imbalance in the AE dataset as a part of ML data preprocessing. Table 3 depicts the hyperparameters for training the mentioned classification models, Figure 8 illustrates graphically the ML experiment.

## 4. Results and Discussion

The results are segregated into various subsections based on the conducted coating experiment and the subsequent ML experiment to gain insights into each effective variable easily.

### 4.1. CD Examination

**Figure 9:**
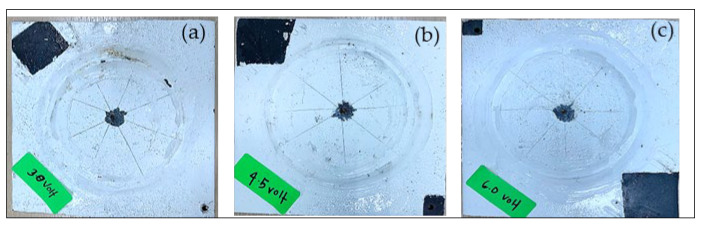
Test panels after exposed at various charging voltages: (**a**) 3.0 V, (**b**) 4.5 V and (**c**) 6.0 V.

A visual examination was immediately performed at the end of the CD test (Figure 9). To obtain the disbonded radius of the defect during the experiment, we made a series of radial cuts across the panel passing through the center of the artificial defect (drill hole) at approximately 45° using a utility knife. Next, we attempted to peel the disbonded coating from the substrate, which delaminated radially outward from the defect site. The extent of the disbondment radius along each cut from the edge of the holiday was calculated as an average value (Table 4).

The average disbonded area for all test panels was below 3 cm^2^. The thinning of the coating in the CD cell was caused by chemical attacks resulting from the electrochemical process driven by the electrical current [15]. Furthermore, the transport of water and ionic species through the coating and dissolution of the oxides directly caused the coating delamination from exposure to a direct current supply during testing. The influence of electrode potential (which refers to changes due to the current supplied) greatly changed the behavior of coating disbondment. In this reaction, hydrogen evolution was the dominant reaction on the test panel, and it limited the transport of oxygen to the exposed metallic surface.

Table 4 also indicates that the disbonded area increased with the increase in electrical current supply. When the electrical current supply was increased, the cathodic potential of the panel also increased. Thus, hydrogen evolution became more active, and the disbondment of coating activities became aggressive.

### 4.2. ML Experiment

**Figure 10:**
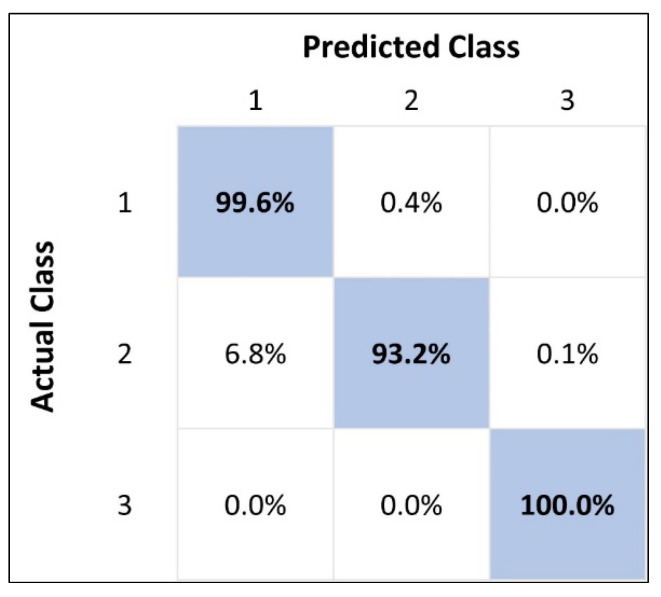
Confusion matrix of the best performing decision forest classification model.

Table 5 depicts the results of the evaluation of five state-of-the-art ML classification models on the AE dataset in failure severity prediction. The best values are presented in bold. The results are presented in terms of standard classification metrics, namely, accuracy, precision, recall, and F1-score, which are defined as follows:

(1) $$Accuracy=\frac{\left(TP+TN\right)\;}{\left(TP+TN+FP+FN\right)}$$

(2) $$Precision=\frac{TP}{\left(TP+FP\right)}$$

(3) $$Recall=\frac{TP}{\left(TP+FP\right)}$$

(4) $$F1-score=\frac{2TP}{\left(2TP+FP+FN\right)}$$

where *TP*, *TN*, *FP*, and *FN* represent the true-positive, true-negative, false-positive, and false-negative entries of the classification confusion matrix, respectively. Figure 10 depicts the confusion matrix for the best-performing decision forest.

Table 5 reveals that decision forests achieved the most promising results on accuracy, precision, recall, and F1-score of 99.4849%, 98.7695%, 97.5838%, and 98.1730%, respectively, followed by decision jungle and logistic regression. Among the evaluated classification models, the decision-tree-based models (forest and jungle classifiers) performed better than the others.

We believe this is because these models are ensemble models and can iteratively improvise the output and build upon an improved model for each run. Hence, these models yield better outputs in comparison with other classification models. Between the decision forest and decision jungle classifiers, the former yield better results for coating disbondment failure severity level prediction due to the inherent nature of the AE dataset. The AE dataset had linear decision boundaries, which eased classification for the decision forest model compared with the decision jungle model, which operates well for nonlinear decision boundaries.

## 5. Conclusions and Future Work

The current work investigated the correlation between AE features and coating disbondment events due to CD. The work conclusions are summarized as follows:

- With the help of the experimental tests, the correlation between the disbonded area during the CD test and AE features can be directly correlated.
- ML can be used to predict the failure severity levels of coating disbondment events from the AE feature dataset.
- The decision forest classification model outperformed the other evaluated state-of-the-art classification models in terms of standard classification metrics for failure severity predictions due to their ensemble capability and the linear decision boundaries of the AE dataset.

The results of this work can be used in the future to identify the most important features involved in coating breakdown and/or failure mechanisms and extended to clustering analysis. Thus, a study of the vital features of ML-based coating disbondment classification via various feature selection techniques can also be performed as a part of future work. Moreover, we conclude that ML is a potential approach that has an important influence on inspection services. Thus, the proposed ML-based coating disbondment classification approach can be extended to other types of coatings, and the formulated AE dataset can be utilized for future ML-based coating disbondment research. Apart from the formulated AE sensor dataset, an image-based dataset for different coating failures, such as blistering, pin holes, chalking, and delamination, can be formulated in the future, and computer vision techniques, such as convolutional neural networks, can be used on image-based datasets for failure classification.

## Figures and Tables

**Table 1:** Specification of AE sensor.

S.#	Criteria	AE Sensor
1	Type	R1.5I
2	Resonant frequency	14 kHz
3	Operating temperature	−35 to 75 °C
4	Structure	Isolated
5	Weight	130 g
6	Preamp (built in)	40 dB

**Table 2:** The extracted AE features.

S.#	Features	Description	Unit
1	Time	The time when the AE signal was taken.	Microsecond (µs)
2	Rise time	The time the first threshold crosses to the highest voltage point on the AE waveform.	Microsecond (µs)
3	Amplitude	The highest voltage in the AE waveform.	Millivolt (mV)
4	Duration	The time from the first to the last threshold crossing.	µs
5	Count	The number of times the signal crosses the detection threshold.	-
6	Counts to peak	The number of thresholds crossing from the first to the highest voltage point on the waveform.	-
7	Count rate	The count divided by time.	-
8	Root mean square (RMS)	The RMS voltage during a period based on a software-programmable time. Constant refers to the input to the signal-processing board.	Volt (V)
9	Reverberation frequency (R-Frequency)	Counts–Counts to peak divided by the Duration–Rise time.	Kilohertz (kHz)
10	Initiation frequency (I-Frequency)	Counts to peak divided by the Rise time.	Kilohertz (kHz)
11	Average frequency (A-Frequency)	Counts divided by the Duration, divided by 1000.	Kilohertz (kHz)
12	Absolute energy	The time integral of the square of the signal voltage at the sensor before any amplification is divided by a 10-kiloohm (kΩ) impedance.	Attojoule (aJ)
13	Average signal level (ASL)	RMS, converted to the dB scale.	dB
14	Energy	The time integral of the absolute signal voltage. The reported magnitude depends on the value selected for energy reference gain and is proportional to the signal strength.	Joule (J)
15	Signal strength	Time integral of the absolute signal voltage.	Picovolt-second (pVs)

Note: none of this is a spectral domain calculation, but a calculation from time domain features.

**Table 3:** Hyperparameters configuration for ML experiment for the failure severity level prediction of coating disbondment.

S.#	ML Model	Hyperparameter Configuration
1	Neural network	Fully connected
		Number of layers = 3 Input layer = 15 nodes (AE feature dataset) Hidden layer = 100 nodes Output layer = 3 nodes (failure classes)
		Activation function = Softmax
		Learning rate = 0.1
		Learning iterations = 100
		Initial learning weight = 0.1
		Momentum = 0
2	Decision jungle	Resampling method = bagging
		Number of decision DAGs = 8
		Maximum depth of the decision DAGs = 32
		Maximum width of the decision DAGs = 128
		Number of optimization steps per decision DAG layer = 2048
3	Decision forest	Resampling method = bagging
		Number of decision trees = 8
		Maximum depth of the decision trees = 32
		Number of random splits per node = 128
		Minimum number of samples per leaf node = 1
4	SVM	Number of iterations = 1
		Lambda = 0.001 Kernel = linear
5	Logistic regression	Optimization tolerance = 1 10^−7^
		L1 regularization weight = 1
		L2 regularization weight = 1
		Memory size for L-BFGS = 20

**Table 4:** The average value of disbonded area of test panel.

S.#	Sample	Applied Voltage (V)	Disbonded Area (cm^2^)
1	S1	3.0	1.83
2	S2	4.5	2.27
3	S3	6.0	2.54

**Table 5:** ML experiment results for the failure severity level prediction of coating disbondment.

S.#	ML Model	Accuracy	Precision	Recall	*F*1-Score
1	Neural network	98.4085	95.0842	93.4875	94.2790
2	Decision jungle	99.2309	98.1902	96.3262	97.2493
3	Decision forest	**99.4849**	**98.7695**	**97.5838**	**98.1730**
4	SVM	96.9538	94.2242	83.1240	88.3267
5	Logistic regression	97.1891	92.7320	86.0707	89.2773

## Data Availability

The AE ML dataset and the ML experiment details are available on request.

## Abbreviations

The following abbreviations are used in this manuscript:

ADC	Analogue-to-digital converter
AE	Acoustic emission
A-Frequency	Average frequency
aJ	Attojoule
ASL	Average signal level
CD	Cathodic disbondment
CNN	Convolutional neural network
CP	Cathodic protection
DAQ	Data acquisition system
dB	Decibel
FP	False positive
FN	False negative
I-Frequency	Initiation frequency
J	Joule
kHz	Kilohertz
kΩ	Kiloohm
ML	Machine learning
NDT	Nondestructive testing
PAC	Physical Acoustics Corporation
PLB	Pencil lead break test
pV	Picovolt
R-Frequency	Reverberation frequency
RMS	Root mean square
SHM	Structural health monitoring
SVM	Support vector machines
TP	True positive
TN	True negative
V	Volt
